# Late Migration of a Bryan Cervical Total Disc Replacement Resulting in Progressive Cervical Myelopathy: A Case Report

**DOI:** 10.7759/cureus.105845

**Published:** 2026-03-25

**Authors:** Jorge Perera, Jacob Pearson, Wael Ghacham

**Affiliations:** 1 Orthopedic Surgery, Good Samaritan Regional Medical Center, Corvallis, USA; 2 Orthopedics, Good Samaritan Regional Medical Center, Corvallis, USA; 3 Spine Surgery, Good Samaritan Regional Medical Center, Corvallis, USA

**Keywords:** anterior cervical discectomy and fusion (acdf), cervical artificial total disc replacement, compression cervical myelopathy, orthopaedic complications orthopedic surgery, orthopedic spine surgery

## Abstract

Cervical disc arthroplasty (CDA) is a well-established motion-preserving treatment for cervical degenerative disc disease. The Bryan® Cervical Disc is among the most widely used implants with supportive long-term outcome data; however, late implant migration is a rare occurrence and can result in significant neurologic compromise. A female in her mid-50s with a history of C5-6 Bryan Cervical Disc arthroplasty performed 20 years prior presented with progressive cervical myelopathy. She reported a fall from a horse two years before evaluation. Neurologic examination demonstrated bilateral upper-extremity weakness distal to the C5-6 motor distribution, hyperreflexia, and positive bilateral Hoffmann sign, Spurling test, and Lhermitte's sign without clonus. Cervical radiographs with flexion-extension views demonstrated posterior migration of the C5-6 prosthesis, concerning for canal encroachment. The patient underwent removal of the arthroplasty device with C5-6 anterior cervical fusion, resulting in successful decompression and clinical improvement. Very late posterior migration of a Bryan Cervical Disc arthroplasty can present decades after implantation and may cause progressive cervical myelopathy. New or worsening myelopathic findings in patients with prior cervical disc replacement should prompt urgent evaluation for delayed mechanical failure, including implant migration. Implant removal and conversion to fusion can provide definitive decompression and stabilization with favorable outcomes.

## Introduction

Cervical disc arthroplasty (CDA) is an established motion-preserving surgical option for appropriately selected patients with symptomatic cervical degenerative disc disease. In the literature, CDA has most commonly been indicated for carefully selected patients with cervical disc herniation or spondylosis causing radiculopathy and/or myelopathy at one or two levels after failure of nonoperative treatment, particularly when there is preserved segmental motion, maintained disc height, and no substantial instability, severe facet arthropathy, or advanced spondylotic collapse [1-3]. Outcomes across the CDA literature, including systematic reviews and meta-analyses, generally support durable clinical improvement in pain and function [1]. Contemporary reviews describe CDA as an effective alternative to fusion in selected single-level pathology and, in certain contexts, as part of multilevel or hybrid constructs [2,3]. The Bryan® Cervical Disc, manufactured in Mérignac, France, by the company Companion Spine France SAS, is among the most studied cervical arthroplasty devices, with multiple cohorts demonstrating sustained patient-reported improvement through mid- and longer-term follow-up [4,5]. Nevertheless, CDA is associated with recognized complications and failure mechanisms, including heterotopic ossification, subsidence, osteolysis, device wear, and implant migration, some of which require revision surgery [6]. Bryan-specific complications have been reported as well, emphasizing that mechanical dysfunction can occur even with otherwise favorable long-term outcomes [7].

Migration is a particularly consequential complication because it may lead to neural element compression and progressive myelopathy. Traumatic migration of the Bryan Cervical Disc has been described, and traumatic loosening has also been associated with complications such as metallosis in isolated reports [8,9]. Device design and material characteristics vary among cervical disc implants and may influence long-term mechanical behavior and interface loading, with newer viscoelastic concepts intended to better replicate physiological motion [10]. Other CDA constructs from different manufacturers, including ball-and-socket, semi-constrained, and mobile-core designs, have likewise demonstrated generally favorable clinical outcomes, although reported complication profiles may differ by implant design, fixation method, and bearing surface [2,3,6,10]. Compared with anterior cervical discectomy and fusion (ACDF), CDA has been associated with preservation of segmental motion and, in some studies, lower rates of adjacent segment degeneration or reoperation, while fusion remains a reliable and well-established treatment with predictable decompression and stabilization [1-3]. In the case of the Bryan Disc specifically, reported construct-related complications include migration, loosening, wear-related problems, and metallosis, highlighting that this motion-preserving device remains susceptible to mechanical failure despite otherwise durable long-term outcomes [6-9]. With regards to long term outcome data, implant migration appears to remain a rare occurrence. One long-term series reported posterior migration in one of 27 operated segments, although when it occurs, it may result in significant neurologic compromise [11].

We report a female in her mid-50s who developed progressive cervical myelopathy associated with posterior migration of a C5-6 Bryan Cervical Disc arthroplasty, presenting 20 years after the index procedure, following a fall from a horse two years prior to evaluation. Although implant migration has been rarely reported in long-term follow-up of the Bryan Cervical Disc, this case appears to represent an exceptionally delayed symptomatic posterior migration occurring two decades after implantation. This unusually late presentation highlights the need to consider delayed mechanical failure in patients who develop new myelopathic signs long after CDA.

## Case presentation

**Figure 1:**
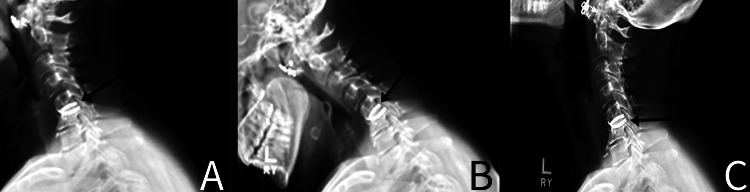
Initial presentation views of the cervical spine A: Lateral neutral view of the cervical spine (arrow demonstrating posterior displacement). B: Lateral flexion view of the cervical spine (arrow demonstrating posterior displacement). C: Lateral extension view of the cervical spine (arrow demonstrating posterior displacement)

**Figure 2:**
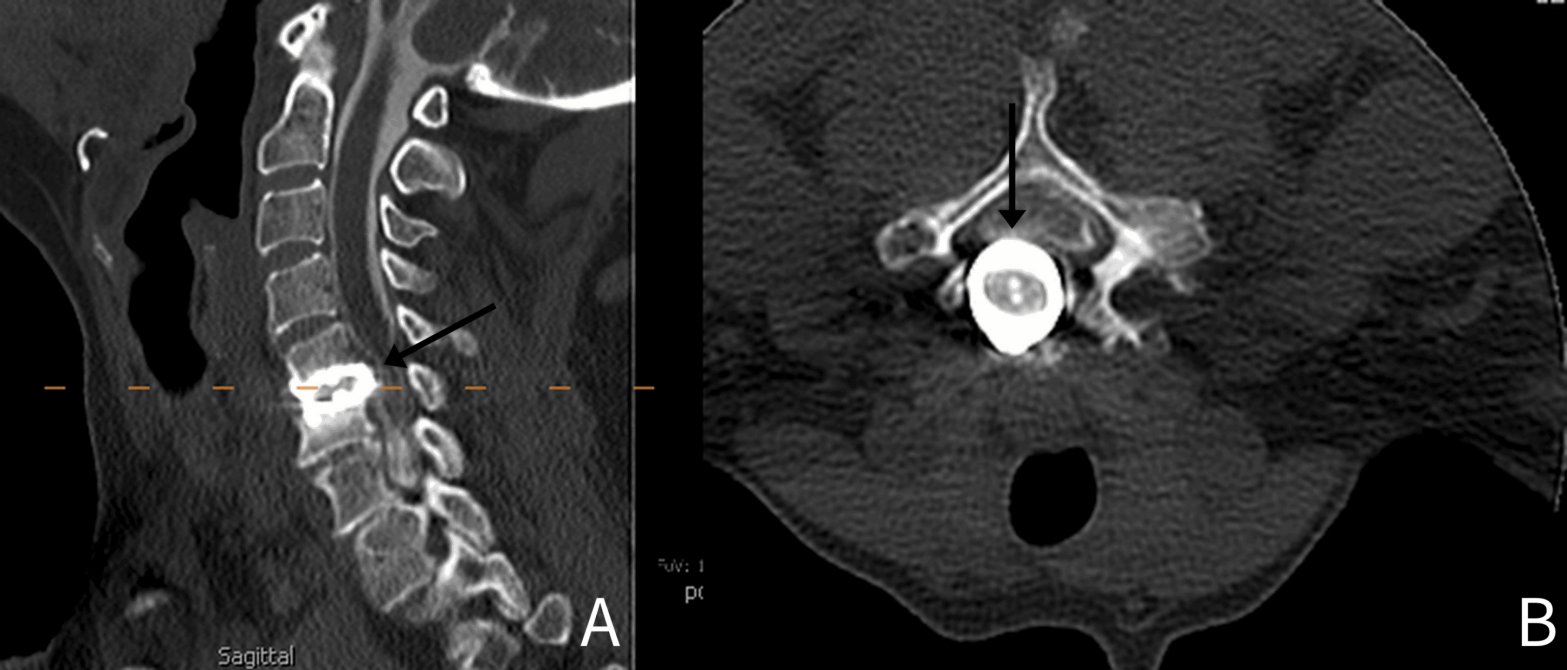
Initial presentation CT myelogram demonstrating posterior migration of the implant A: Sagittal view of the cervical spine (arrow demonstrating posterior displacement of implant). B: Axial view of the cervical spine at the C5-6 level (arrow demonstrating posterior displacement of the implant)

We present the case of a female in her mid-50s with a past medical history significant for gastric bypass surgery, depression, and a history of smoking 10 cigarettes daily, who presented to the clinic with bilateral upper extremity weakness, numbness, and “shock”-like pain for the past two years. The patient underwent a C5-6 total disc replacement surgery in 2003, and she recovered well with no issues postoperatively. Two years prior to evaluation, the patient fell off her horse, which began the return of her myelopathic symptoms. It is unclear as to whether this trauma was the primary precipitating factor for implant migration or if it may have contributed to progressive loosening over time. However, according to the patient, after that incident, her symptoms became increasingly worse, especially in the year leading up to presentation at the clinic. She tried extensive conservative treatment, including activity modification, over-the-counter pain medications, and corticosteroid treatment, all of which had not provided any adequate relief. Prior to arrival, the patient completed a recent cervical spine X-ray (Figure 1), as well as a computed tomography (CT) myelogram (Figure 2). She also had labs drawn, including CBC and CRP, which were within normal limits. Of note, the patient states that she had been tobacco and nicotine-free for two years prior to presentation.

The patient presented to our clinic alert and oriented, with no acute distress, and stable vital signs. There was a well-healed incision with approximated skin edges and no erythema, discoloration, or drainage located on the right side of the patient's anterior neck around the C5-6 level. There was some tenderness to palpation along the cervical spinal musculature, as well as significant pain with flexion, extension, and rotation of the neck. On motor strength testing, there was significant weakness bilaterally distal to the C5-6 motor distribution, with 4/5 grading of biceps, triceps, wrist extensor, and abductor pollicis brevis strength. There was diminished sensation noted over the C5-7 dermatomal distributions. The patient demonstrated hyperreflexia with biceps, brachioradialis, and triceps reflex testing. Hoffman's sign was present and positive bilaterally, as well as positive Spurling testing and Lhermitte's sign. No clonus was appreciated on exam.

At this point, the patient's clinical examination was pathognomonic for cervical myelopathy. An independent review of diagnostic imaging was performed in the clinic and reviewed by both the radiologist and spine surgeon. Both expert interpretations were concordant with each other and the patient's symptomatology. X-rays of the cervical spine, including flexion and extension stress views, demonstrated a C5-6 disc prosthesis that extended 3 mm proud to the posterior cortex of the vertebral body, likely into the cervical spinal canal (Figure 1). This measurement was obtained on the neutral lateral view, measuring the extent of the implant from the posterior cortex of the C5 vertebral body. No abnormal motion is noted on flexion or extension views.

CT myelogram of the cervical spine demonstrated a similar posterior projection of the artificial disc positioned at C5-6 disc space with what appears to be osteolysis across the inferior endplate of the C5 vertebral body and concomitant irregularity along the superior endplate of C6 (Figure 2). The axial view of the CT provides the most noticeable evidence of canal compromise, with demonstration of posterior implant migration and effacement of the ventral border of the spinal cord.

With this patient's physical examination, X-ray imaging, and CT myelogram, she was diagnosed with cervical central stenosis at C5-6 with myelopathy and C5-6 total disc replacement failure with hardware loosening. MRI was determined not to be clinically necessary in this case. The Bryan Cervical Disc is not considered MRI-contraindicated, but artifact from CDA can limit assessment of the operated level. Therefore, CT myelography was used to better characterize cord compression and posterior implant migration.

At this juncture, it was explained to the patient in detail that her myelopathic symptoms would continue to worsen over time if surgical intervention were not pursued. We felt that hardware removal was necessary, and therefore an anterior-based approach was preferred as opposed to the consideration of a posterior cervical decompression surgical option. In this same manner, as the patient had evidence of osteolysis of the inferior endplate of C5 and superior endplate of C6 with an already failed previous arthroplasty procedure, we felt that it was in the patient's best interest to proceed with a fusion surgery as opposed to the alternative. Our recommendation, therefore, was to proceed with urgent hardware removal followed by anterior cervical decompression and fusion at the C5-6 level. We also counseled the patient on the need for an ENT evaluation prior to surgery, as we planned on proceeding from a left-sided anterior approach, and she previously had a right-sided approach. She agreed with the plan and had no further questions at that time.

**Figure 3:**
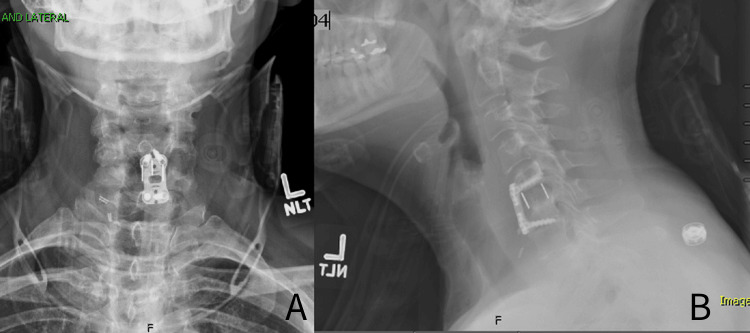
Postoperative day one X-ray views of the cervical spine A: AP view of the cervical spine showing postoperative changes after hardware removal and anterior cervical discectomy and fusion (ACDF) at the C5-6 level. B: Lateral view of the cervical spine showing postoperative changes after hardware removal and ACDF at the C5-6 level

Approximately one month after initial presentation to our clinic, the patient underwent successful C5-6 hardware removal and anterior cervical decompression and fusion at the C5-6 level. There were no complications encountered during the less than two-hour surgery. Blood loss was minimal, and aside from mild-to-moderate subchondral osteolysis both superiorly and inferiorly surrounding the implant, there were no unexpected findings. Neuromonitoring was used throughout the case, and a JP drain was placed prior to closure of the wound. The drain was removed, and X-ray imaging (Figure 3) was completed on postoperative day one, at which point she was medically deemed stable for discharge. She was discharged from the hospital with an Aspen collar in place.

Upon discharge, the patient was weight-bearing as tolerated and was to follow up in our clinic at two weeks postoperatively for a wound check and re-evaluation. At this first appointment, the patient was doing well and stated that all her preoperative myelopathic symptoms had been resolved. She demonstrated 5/5 strength in all muscle groups of the bilateral upper extremities. At that juncture, she was advanced to activity as tolerated but was to avoid high-impact activities or lifting anything greater than 10-15 lbs. She was told to remain in an Aspen collar while active until at least six weeks postoperatively.

Six weeks after the initial follow-up, the patient was still feeling much improved and very pleased with the outcome of the surgery. Due to her work schedule, she had not started physical therapy but remained consistent with a home exercise and stretching program. Physical exam results remained promising, with complete cessation of Hoffman's sign and hyperreflexia. At this time, the patient's limitations were further advanced to cardiovascular exercises and gradual return to resistance training with a planned follow-up in three months.

**Figure 4:**
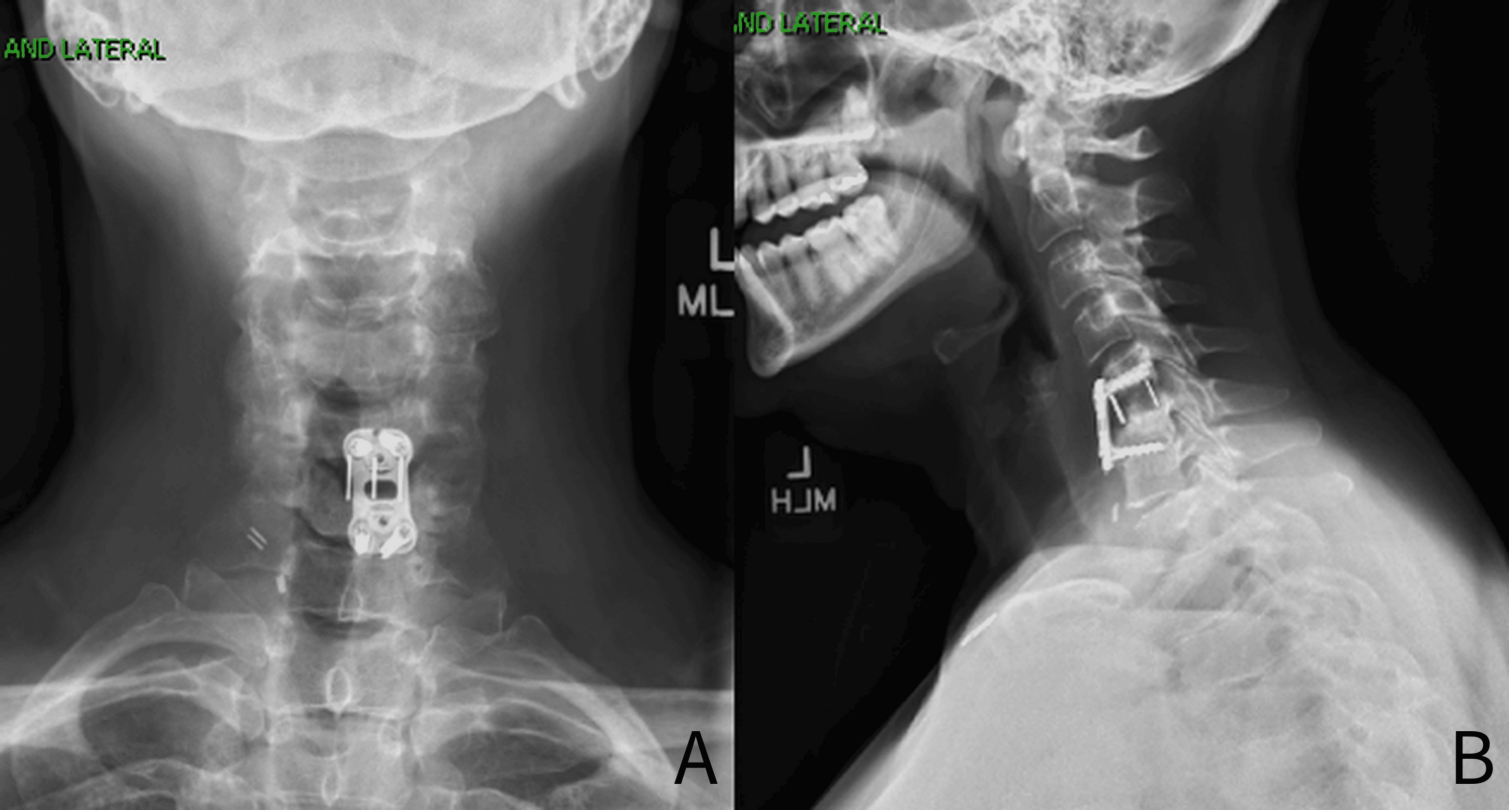
Five-month postoperative X-ray views of the cervical spine A: AP view of the cervical spine demonstrating a well-positioned anterior cervical discectomy and fusion (ACDF) plate and screw construct with no migration as compared to previous views. B: Lateral view of the cervical spine demonstrating a well positioned ACDF plate and screw construct with no migration as compared to previous views

The patient was seen again approximately five months postoperatively and continued to do well. X-ray imaging was taken in the clinic, which demonstrated caudally placed screws into the C6 vertebrae, but this was stable as compared to previous views (Figure 4). There was some bone formation noted in the C5-6 intervertebral space. The patient was allowed to return to horse riding but was still cautioned to avoid high-impact activity as there was still time for complete healing of her fusion procedure.

At this point, it was explained to the patient that we would like to take one final set of X-rays one year out from surgery, and a phone call visit could be scheduled to go over the results. She agreed with the plan but was lost to follow up.

## Discussion

This case highlights a rare but clinically important late failure mode of CDA: posterior migration of a Bryan Cervical Disc replacement resulting in progressive cervical myelopathy, occurring two decades after implantation. Compared with previously reported Bryan disc migration-related complications, this case appears notable for both the extreme delay in presentation and the presence of progressive myelopathy from posterior canal encroachment. Prior reports have described trauma-associated migration or loosening of the Bryan device, but those cases emphasize mechanical failure in a more acute post-traumatic setting rather than symptomatic posterior migration presenting decades after implantation [8,9]. This distinction underscores the unusual timing and neurologic severity of the present case. CDA generally demonstrates favorable outcomes in systematic reviews and meta-analyses, and the Bryan Disc has published mid- and long-term follow-up supporting sustained clinical improvement, but uncommon mechanical complications can still occur and may carry substantial neurologic consequences [1-5]. Reviews of CDA complications emphasize that failure patterns include device wear, loosening, subsidence, and migration, many of which are encountered in the early to intermediate postoperative period, whereas very late mechanical failures appear to be uncommon. In a recent revision-focused analysis, migration predominated in the early and intermediate phases, while later failures were more often related to neck pain, osteolysis, or heterotopic ossification [6]. This contrasts with the present case, in which symptomatic posterior migration occurred 20 years after implantation, far beyond the time horizon of most reported migration events and beyond the major Bryan long-term follow-up series, which reported only rare migration at eight years and no migration at four years. Bryan-specific complication reporting similarly supports that mechanical dysfunction, although infrequent, remains a recognized entity [7].

Previously reported Bryan disc migration-related complications have generally focused on trauma-associated mechanical failure, implant loosening, or revision management, rather than an exceptionally delayed presentation with progressive cervical myelopathy [6-9]. Compared with those reports, the present case is unusual because posterior migration produced canal compromise and symptomatic myelopathy 20 years after implantation. This distinction highlights both the rarity of the presentation and the importance of considering delayed implant-related pathology even decades after CDA.

In prior reviews of CDA failure, implant migration is considered clinically significant because even limited displacement may indicate mechanical instability and may necessitate revision when associated with neurologic compromise [6,7]. In the present case, the significance lies not in the radiographic measurement itself, but in the fact that posterior migration was associated with progressive myelopathy, supporting the need for surgical decompression and stabilization.

Most revision-driving mechanical problems after CDA are encountered earlier in follow-up, when issues related to device seating, endplate integrity, and early biologic response may influence stability [2,6]. In contrast, our patient became symptomatic 20 years after Bryan Disc implantation. This is an exceptionally delayed presentation relative to the major Bryan follow-up series, which demonstrates durable outcomes over long-term surveillance but cannot fully capture very rare complications on a multi-decade horizon [4,5]. The history of a fall from a horse two years before presentation raises the possibility that trauma contributed to implant destabilization, loosening, or delayed interface compromise. Traumatic migration of the Bryan Cervical Disc has been documented in literature, supporting trauma as a plausible precipitating factor in certain cases [8]. Similarly, traumatic loosening with subsequent metallosis has been reported, reinforcing that trauma-related mechanical disruption can lead to delayed pathologic consequences [9]. Our case extends these observations by suggesting that trauma may initiate a mechanical cascade that presents clinically much later as progressive myelopathy.

Disc replacement devices differ in constraint, materials, and intended kinematics, with viscoelastic design concepts intended to better approximate physiologic motion and load sharing [10]. Although the Bryan disc is not categorized as a newer viscoelastic implant, it remains relevant that implant design and long-term load transfer may influence the likelihood and nature of delayed mechanical complications. As CDA utilization grows and longer-term follow-up becomes more common, rare late events such as the one described here may become increasingly recognized [10]. When posterior migration produces canal compromise and progressive myelopathy, revision surgery is typically indicated to remove the compressive source and restore stability. Reviews of CDA failure highlight that hardware removal and conversion to fusion is a common revision pathway in the setting of mechanical failure [6,7]. In this case, the patient underwent removal of the Bryan Disc prosthesis, followed by C5-6 ACDF, resulting in a successful clinical outcome. The favorable response supports device removal and fusion as an effective treatment strategy for symptomatic migration producing neurologic compromise. Existing literature supports overall durable outcomes after Bryan Disc arthroplasty while acknowledging that mechanical complications can occur [4-7]. Case-based reports demonstrate that trauma can be associated with migration or loosening-related complications [8,9]. The present case is distinctive because it represents symptomatic posterior migration with myelopathy occurring 20 years after implantation, a timeframe that appears to be exceptionally rare in published reports. The practical implication is that CDA remains relevant in history even decades after implantation, and progressive neurologic symptoms should prompt urgent assessment for delayed mechanical failure.

This case report has several limitations. As a single case report, definitive causation and generalizability are limited. While trauma is a plausible contributing factor, the precise mechanism of multi-decade delayed migration cannot be established. In this retrospective single case review, standardized outcome scores, as well as additional operative metrics, were not obtained, which therefore lessens the comparability of this case report. In addition, long-term postoperative follow-up was not obtained as the patient was not seen again after her five-month postoperative appointment. Nonetheless, the case provides an important clinical reminder regarding late arthroplasty-related pathology and the need for rapid evaluation when myelopathic signs develop.

## Conclusions

Posterior migration of a Bryan Cervical Disc arthroplasty is an uncommon complication. Symptomatic migration presenting two decades after implantation is either exceptionally rare or underreported, as there is little data in the literature with evidence of this complication so many years after the index procedure. This case demonstrates that delayed mechanical failure can manifest as progressive cervical myelopathy. In patients with a history of cervical disc arthroplasty who develop new myelopathic symptoms, clinicians should maintain a high index of suspicion for implant-related canal compromise and obtain timely imaging. When posterior migration produces spinal canal encroachment and neurologic deterioration, prompt removal and conversion to fusion can provide definitive decompression and stabilization with good clinical outcoms.
